# High-Fidelity Simulation-Based Education: Description of an Original Crisis Resource Management and Sedation Learning for Dental Surgeons

**DOI:** 10.3390/ejihpe12020008

**Published:** 2022-01-24

**Authors:** Issam Tanoubi, Roger Perron, Marie-Ève Bélanger, Mihai Georgescu, Arnaud Robitaille, Pierre Drolet

**Affiliations:** Medical Simulation Centre, Centre D’apprentissage des Attitudes et Habiletés Cliniques (CAAHC), Université de Montréal, Pavillon Roger-Gaudry, 2900, Boulevard Édouard-Montpetit, 8e étage, Local N-805, Montréal, QC H3T 1J4, Canada; roger.perron@umontreal.ca (R.P.); marie-eve.belanger.7@umontreal.ca (M.-È.B.); leonida-mihai.georgescu@umontreal.ca (M.G.); arnaud.robitaille@umontreal.ca (A.R.); pierre.drolet@umontreal.ca (P.D.)

**Keywords:** high-fidelity simulation, crisis resource management, sedation

## Abstract

Dental surgery includes invasive procedures performed under sedation or monitored anesthesia care (MAC). It is associated with respiratory risks, resulting in death or neurological sequelae without prompt and appropriate management. Management of airway complications also implies mastering crisis resource management (CRM) principles, essentially non-technical skills to improve patient safety. In response to the need to enhance patient safety and to securely perform surgical procedures outside the operating room due to reduced surgical activity during the worldwide spread of the COVID-19 pandemic, we realized, in our simulation center, a course based on high fidelity simulation to teach procedural sedation and management of related complications. The simulation center accredited this educational program as a continuing professional development formation. The course includes technical skills practice, theoretical presentation, and mastering non-technical skills related to CRM principles. This brief report describes a relatively innovative teaching technique in dentistry, highlights its interest, and reports the subjective opinion of learners as to the pedagogical and professional impact of this training. A learner’s satisfaction survey supports the utility of our sedation and CRM programs. A high degree of satisfaction and perceived value reflect robust learners’ engagement. All medical specialties should encourage high-fidelity simulation continuing professional development courses that incorporate technical skills and crisis management principles.

## 1. Introduction

Dentistry and maxillofacial surgery include minimally invasive surgical procedures, usually performed outside the operating room under sedation or monitored anesthesia care (MAC). Most of the time, practitioners provide MAC without additional human resources specific for patient monitoring. Complications related to MAC are rare but can lead to severe consequences without early and adequate management. The suboptimal control of sedation-related complications is usually associated with a lack of resources. In the context of dental and maxillofacial surgery outside the operating room, the missing resources are human (medical specialists, health personnel) and practical and theoretical cognitive order. As frequently recommended, theoretical and practical learning of safe administration and management of complications related to MAC to non-anesthesiologist seems mandatory [1].

The use of high-fidelity simulation as a teaching tool has already demonstrated its effectiveness, particularly in the crisis resource management (CRM) principles field [2,3].

We have developed a learning program of MAC administration at our simulation center, management of complications related to sedation, and CRM learning, designed for dental and maxillofacial surgeons. The program is mainly based on high-fidelity simulation and a multimodal approach with low-fidelity simulation for technical skills and a one-hour presentation for the theoretical principles. Several doctors have participated in this program and have expressed high satisfaction from their learning experience.

This brief report describes our relatively innovative simulation-based teaching technique in dentistry, highlights its interest, and reports the subjective opinion of learners as to the pedagogical and professional impact of this training. The aim of the report is mainly descriptive of the postgraduate training. This study was a first step to assess the acceptability and satisfaction of learners. It helps us decide whether to continue the course. The assessment of this simulation-based course through the learners’ experience offers a low scientific evidence level as to the professional or pedagogical effectiveness, that we plan to evaluate through subsequent studies

## 2. Materials and Methods

### 2.1. Simulation-Based Sedation Program

This educational program has received our university-affiliated anesthesiology department’s research and development grant. All participants (trainees) provided written informed consent to publish a description of their training program and their satisfaction. This study was performed following the Declaration of Helsinki and the human subject’s protection standards in research.

#### 2.1.1. Need Assessment 

**Figure 1:**
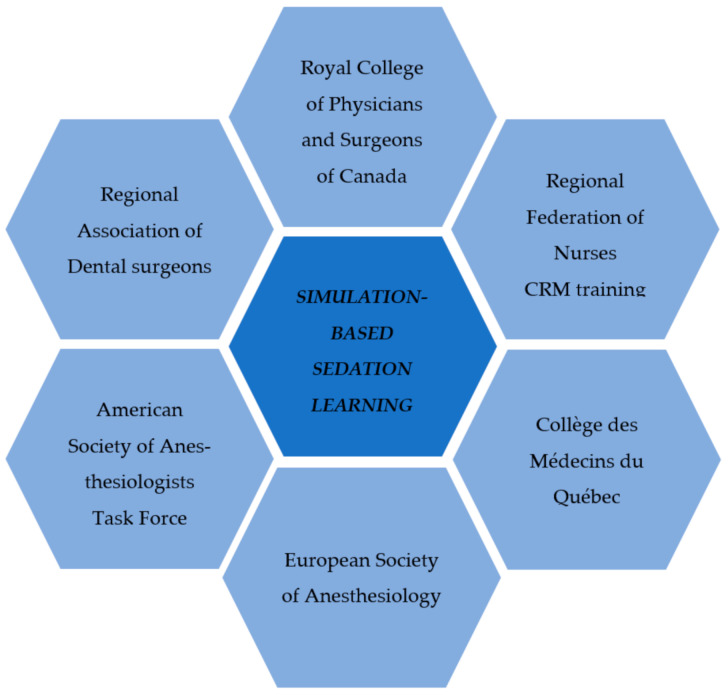
Simulation-based need assessment sources. CRM: Crisis Resource Management.

The need to learn the safe sedation practices and CRM principles were Figure 1 initially felt and expressed by many members of our Regional Association of Dental Surgeons [4]. They were concerned about the safety of sedation outside the operating room, especially when practicing invasive surgical procedures requiring higher sedation levels. The necessity to offer this kind of training to the surgeon assistant (nurse or respiratory therapist) was also objectified [5]. Several expert committees also dictated the relevance of such educational programs [1,6]. They recommend that the practitioner of procedural sedation have prior training in sedation administration and management of its respiratory complications. The same training is also mandatory for the practitioner assistant. In addition, our Regional College of Physicians and our Regional Federation of Nurses require that their members have prior sedation management and CRM learning [7,8].

#### 2.1.2. Educational Objectives

The educational objectives of this program are identical to the recommendations of our Regional College of physicians to enhance safety during procedural sedation [9]. At the end of the course, the practitioner (a) must be able to provide procedural sedation and analgesia, (b) should demonstrate an understanding of the drugs administered, (c) should be able to monitor the patient’s response to the medications given, (d) and develop the skills to intervene in managing all potential complications.

The management of complications related to sedation will rely on the practitioner’s theoretical knowledge, technical skills, and non-technical skills. These non-technical skills are described in the literature as crisis resource management skills. They include a wide range of competencies like being aware of the environment, anticipating and planning, calling for help early, exercising leadership and followership, distributing the workload optimally, mobilizing all available resources, and communicating effectively. If adapted to respiratory complications occurring during a sedation context, these CRM-non-technical skills could be summarized in: identifying distress, calling early for help, requesting name and role of arriving «help» team, ensuring a read-back of statements and requests, attempting and continually evaluating the effectiveness of ventilation, mastering an effective leadership by entrusting technical skills to the appropriate team members, by restating presumptive diagnosis and vital signs and by asking the team for suggestions. 

We have integrated these skills within this program to cover all the cognitive resources needed to support safe sedation.

#### 2.1.3. Learning Methods

The sedation course is high fidelity and simulation-based. It begins with the visit of the high-fidelity mannequin, a SimMan 3G high-fidelity mannequin (http://www.laerdal.com/ca/SimMan3G accessed on 23 January 2022), and a presentation on the various constraints related to simulation. A fiction contract is implicitly signed with participants at the end of this introduction to encourage them to foster in the scenario and offer more realism to the unfolding situation. 

#### 2.1.4. High Fidelity Simulation and Oriented Debriefing

**Figure 2:**
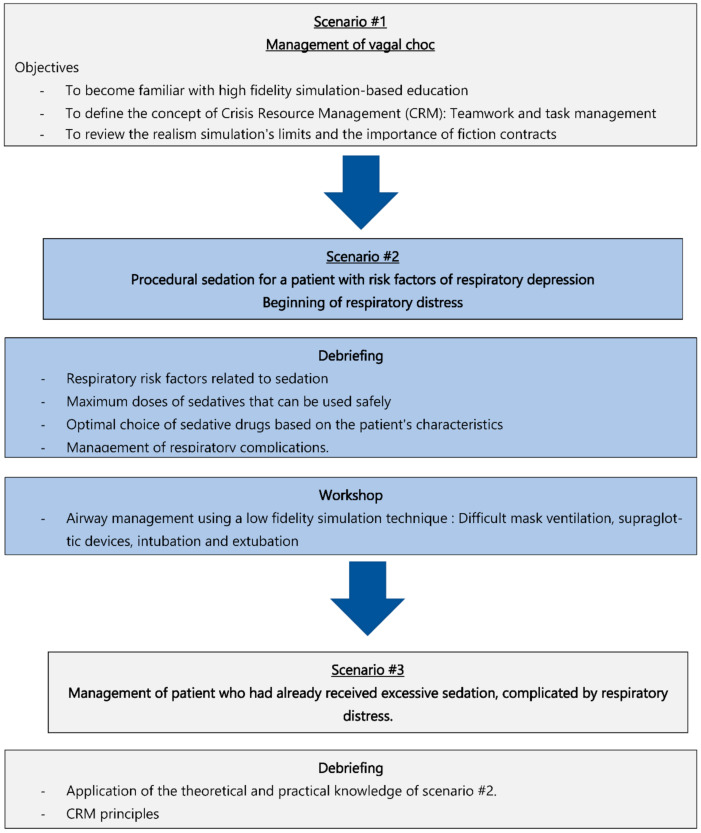
The timeline of the simulation workshop.

The high-fidelity simulation workshop includes three scenarios (Figure 2). The first scenario, managing a patient with vasovagal syncope allows participants to become familiar with the high-fidelity simulation teaching technique. It is followed by a debriefing emphasizing teamwork and task management. This scenario also allows defining the concepts of «crisis» and «situational awareness» and the principles of «crisis resource management (CRM)» During the debriefing, the instructor reviewed the realism simulation’s limits and the importance of fiction contracts.

For the second scenario, participants had to administer procedural sedation for a patient with risk factors of respiratory depression. Following sedation, the patient’s respiratory status will deteriorate with breathing pauses and minor desaturation without respiratory distress. After the scenario, a debriefing is conducted to raise several questions about respiratory risk factors related to sedation, the maximum doses that can be used safely, the choice of sedative drugs based on the patient’s characteristics, and the management of respiratory complications. The debriefing scenario addresses these issues through a theoretical presentation and a practical workshop on the low-fidelity model.

For the last scenario, participants had to manage a patient who had already received excessive sedation, complicated by respiratory distress. The debriefing of the latter scenario is based on the teaching of CRM principles and teaching safe sedation management, including the complications. 

#### 2.1.5. Data Collection

Participants practiced during four days of training (four participants per day). At the end of the training, they were given an evaluation questionnaire on the quality of the training and its impact on their practice. The answers were on a Likert scale from 1 to 4, varying from “completely agree” to “completely disagree”. The questions were (1) The objectives of the activity have been achieved; (2) the scenarios contained realistic situations; (3) the clinical environment is approaching reality; (4) the duration of the debriefing sessions was adequate; (5) I learned during the debriefing sessions; (6) I appreciated my experience; (7) My experience will improve my clinical practice; (8) We used descriptive statistical analysis.

## 3. Results

Sixteen participants took part in the training. All participants were dentists qualified in oral and maxillofacial surgery and members of the Fédération des Dentistes Spécialistes du Québec. The satisfaction survey that follows the workshop shows that more than 75% of participants strongly agreed that the activity’s objectives have been achieved and that the workshop will improve their clinical practice.

**Figure 3:**
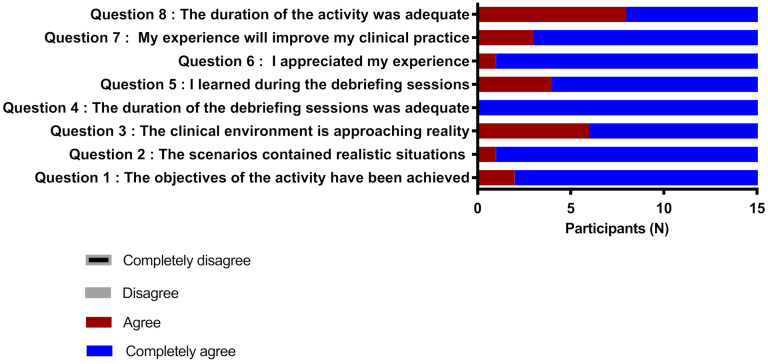
Distribution of results by teaching quality assessment questions. The answers were on a Lickert scale from 1 to 4, varying from “completely agree” to “completely disagree”.

In addition, all the participants agreed or strongly agreed that the scenarios contained realistic situations, and the clinical environment approached reality (Figure 3).

## 4. Discussion

Most practitioners routinely deliver sedation without any specific formation. Sedation is delicate to provide; it ranges from an anxiolysis to general anesthesia, and the patient should be in the perfect state for the procedure. The practitioner must understand the level of sedation needed, identify the patient at risk of complications, be aware of the pharmacology of the drugs administered, and manage the complications. Adverse events related to sedation out of the operating room are concerning and may be prevented by forming non-anesthesiologist physicians. As stated earlier, the need for specific training on sedation is recommended. Simulation-based medical education for learning safe sedation is an excellent way to introduce the concept of crisis resource management. Learners will develop non-technical skills relevant to the management of the complications [9]. The session offers a safe environment to practice technical and non-technical skills. It also includes a lecture on essential airway management, which helps to link theory and practice. This multimodal approach to learning enhances the commitment of learners. The level of satisfaction is very high at the end of the session, and it supports the usefulness of our program. Learners also report that the completion of this module might change their practice. 

Procedural sedation training is highly recommended for non-anesthesiologists [6,10,11]. It requires a mastery of several pharmacological and physiological knowledge that trainers could address through self-learning. On the other hand, managing a respiratory arrest complicating the sedation, a typical clinical crisis, requires a mastery of technical and non-technical CRM skills [12]. These are currently adequately taught through high-fidelity simulation and debriefing [13]. The different educational objectives need the trainer to use various learning techniques during this workshop. Each teaching method responded to the most appropriate educational goal. This objective-based teaching technique was clinically beneficial according to the participants and may impact their current practice. They felt professionally confident after the workshop. The procedural sedation management workshop is currently being repeated for residents of different specialties exposed to sedation management during their curriculum.

Our results regarding trainee satisfaction with the simulation-based sedation course are similar to the data in the literature [9,14,15,16]. Following a high-fidelity simulation workshop, it is usual to find that participants’ self-assessments tend to demonstrate high satisfaction and a strong impression of the effectiveness of the teaching carried out. The self-administered reports allow for social desirability bias. It may raise questions about the degree to which the assessment tools accurately assess the studied constructs [17]. Our results could be due to trainees’ voluntary participation and the innovative nature of the teaching tool [18]. The fact remains that our results would encourage us to continue this learning module, compare it prospectively with another teaching technique and evaluate its impact with indices of patient satisfaction or patient morbidity and mortality.

In addition to ensuring patient safety, this training program could reduce the load on the operating room (OR) in response to the reduction in surgical activity in pandemic periods and make up for the delay in the operating program in the post-pandemic period. We could consider that the minimally invasive maxillofacial surgeries performed outside of the operating under monitored anesthesia care provided by the surgeon, with support staff also professionally trustworthy to manage procedural sedation.

Simulation-based medical education, the learning technique used during this study, was unique and original on two levels. First, high-fidelity simulation has never been used in continuing professional development in dental surgery. In addition, the concept of CRM, already familiar for several medical specialties, was innovative for specialists in dental surgery. Of course, these self-reported evaluations and the level of evidence provided by this evaluation is low. As participant satisfaction is a crucial contributor to individual development in the learning process [19], it can be expected that the CRM principles acquired during the simulation will be applied in the clinical field. On the other hand, in the context of simulation-based medical education, scarce studies show a direct impact of simulation learning on the morbidity or mortality of the patients.

## 5. Conclusions

Our study demonstrated the feasibility and acceptability of a simulation-based procedural sedation learning module for dental surgeons. After assessing the normative needs for sedation learning, we carried out the module according to the deliberate practice principle until a point of professional proficiency in the activity [20]. We included several pedagogical techniques adapted to each educational objective. Our results suggested that the learner felt comfortable and trustworthy with sedation management. Further studies are needed to demonstrate the impact of this teaching on patient satisfaction and safety.

## Data Availability

Data is contained within the article and is available on request from the corresponding author.
